# Nanoparticle-based drug delivery systems: What can they really do *in vivo*?

**DOI:** 10.12688/f1000research.9690.1

**Published:** 2017-05-16

**Authors:** Yi-Feng Wang, Lu Liu, Xue Xue, Xing-Jie Liang

**Affiliations:** 1 Laboratory of Controllable Nanopharmaceuticals, CAS Key Laboratory for Biomedical Effects of Nanomaterials and Nanosafety, CAS Center for Excellence in Nanoscience, National Center for Nanoscience and Technology, Beijing, 100190, China; 2 University of Chinese Academy of Sciences, Beijing, 100049, China

**Keywords:** nanoparticles, drug delivery, efficiency, biocompatilbility

## Abstract

In the past few decades, there has been explosive growth in the construction of nanoparticle-based drug delivery systems (NDDSs), namely nanomedicines, owing to their unique properties compared with traditional drug formulations. However, because of a variety of challenges, few nanomedicines are on sale in the market or undergoing clinical trial at present. Thus, it is essential to look back and re-evaluate what these NDDSs can really do *in vivo*, why nanomedicines are regarded as potential candidates for next-generation drugs, and what the future of nanomedicine is. Here, we focus mainly on the properties of NDDSs that extend blood circulation, enhance penetration into deep tumor tissue, enable controllable release of the payload into the cytoplasm, and overcome multi-drug resistance. We further discuss how to promote the translation of nanomedicines into reality. This review may help to identify the functions of NDDSs that are really necessary before they are designed and to reduce the gap between basic research and clinical application.

## Introduction

With their unique physical and chemical properties as well as their nanoscale effects, nanoparticle-based drug delivery systems (NDDSs) are currently under extensive development for applications in the treatment of diseases such as cardiovascular diseases, infectious diseases, diabetes, and cancer ^1– 3^. It has been reported that most cancers, as malignant diseases, can be suppressed with various NDDSs, including inorganic nanoparticles (NPs) such as metallic NPs and semiconductor nanostructures, organic NPs such as polymer carriers and carbon nanostructures, and hybrid NPs ^4– 6^. Disappointingly, a statistical analysis showed that only 0.7% (median) of the injected dose of the NDDS reached the tumor region in mouse models and this value has not improved in the past 10 years ^7^. There is no doubt that there are systemic effects on the interaction between NDDSs and organisms. From the material perspective, the circulation, biodistribution, internalization, and trafficking of NDDSs are highly dependent on the physicochemical properties of NDDSs such as size, shape, surface chemistry, and material type ^8, 9^. From the biological perspective, NDDSs need to cross or elude a series of complex biological barriers, including opsonization by the mononuclear phagocyte system (MPS), non-specific distribution, interstitial fluid pressure, cellular internalization, and drug efflux pumps, before exerting their therapeutic effect ^7, 10^. In this review, we will focus on discussing the basic functions of NDDSs for cancer therapy and present recently developed strategies for improving the efficacy of NDDSs *in vivo*. Finally, we propose what we need to do to accelerate the translation of nanomedicines in the future.

## Extended blood circulation and accumulation with passive/active targeting

Blood circulation time is an important parameter that affects the therapeutic efficiency and outcome. The fundamental functions of NDDSs are to increase the drug concentration in targeted tissues and to reduce systemic side effects by modulating the pharmacokinetics and biodistribution of the drug payload. In order to increase the drug concentration at tumor sites, longer blood circulation time is vital because it enhances the probability of drug delivery to the tumor without sequestration by the MPS. PEGylation is a widely used approach for prolonging circulation time, and some PEGylated nanomedicines, such as Adagen, Doxil, Macugen, and Pegasys, have been approved for clinical use ^11– 13^. Passive and active targeting are the two alternatives for enhancing the efficiency of NDDS accumulation in tumor tissue. For passive targeting, the theoretical mechanism is the “enhanced permeability and retention” (EPR) effect, which broadly explains why NDDSs can accumulate in tumor tissue through leaky and defective blood vessels ^14, 15^. However, the EPR phenomenon was almost always studied in rapidly growing tumors in mouse models, and it has been suggested that the EPR effect does not work in the clinic, because of a lack of fenestrations in the tumor vessels of patients ^16, 17^. Therefore, the passive targeting strategy needs to be carefully evaluated and further validated in clinical trials. For active targeting, NDDSs mostly use targeting ligands such as antibodies, peptides, and aptamers, which specifically recognize overexpressed “biomarkers” in the tumor microenvironment or the surface of cancer cells after extravasation of the NDDS from blood vessels ^1, 18^; this approach enhances the residence time and the local drug concentration. Strikingly, the targeting ability of NDDSs may be reduced or abolished after the formation of protein corona in the biological milieu ^19^. Regardless of whether they possess passive or active targeting capabilities, the “foreign” NDDSs are directly exposed to the MPS once injected into the body, so the blood circulation time will be decreased.

To overcome the first biological barrier, namely opsonization and sequestration by the MPS, some “invisibility cloak” NDDSs have recently been developed on the basis of biomimetic strategies ^20– 24^. For instance, Zhang *et al*. have reported platelet membrane-cloaked NPs, with biodegradable polymeric NPs inside and immunomodulatory and adhesion antigens on the surface ^23^. The results showed that the cloaked NPs were less efficiently recognized by macrophage-like cells and did not induce complement activation. Also, the cloaked NPs have enhanced adhesion to damaged vasculature and improved therapeutic efficacy compared with uncloaked NPs. The lesson in these cases is that we need to rethink what the body really needs—maybe it is better to work with the body’s natural processes than against them.

## Enhanced penetration depth in tumor tissue

Weak penetration of drug into the deep tissue of solid tumors dramatically attenuates treatment efficacy. Although NDDSs with long circulation periods have the advantage of increased accumulation in the tumor microenvironment after extravasation from blood vessels, penetration into deep tissue remains a challenge because of the high interstitial fluid pressures and poor lymphatic drainage in tumors ^25^. In early studies, thanks to the controllable physicochemical parameters of NDDSs, strategies focused mostly on how to optimize single physicochemical characteristics of NDDSs, such as size, shape, and surface chemistry, to obtain excellent penetration and therapeutic effects ^9, 26– 28^. Recently, a few smart “multistage” NDDSs have been reported to “adapt” biological barriers and improve penetration ^10, 29, 30^. Shen *et al*. have reported an injectable micrometer-sized generator loaded with a pH-sensitive polymeric drug (iNPG-pDox) ^31^. Once released from iNPG after iNPG-pDoxs accumulates at the tumor site, the pDox self-assembly forms NPs inside the tumors and then the pDox NPs are internalized and transported to the perinuclear region. Kohane *et al*. have developed a photoswitchable spiropyran-based NDDS ^32^. Upon irradiation at 365 nm, the size decreased from 103 to 49 nm and the shrinkage enhanced penetration into deep tissue and drug release.

Logically, from a biological perspective, the tumor’s pathophysiological characteristics should be clearly understood so that the NDDS can be designed to effectively penetrate into deep tissue. Tumors are highly heterogeneous and the tumor characteristics depend on the cancer type, pathological state, location, and individual factors such as age, lifestyle, and genetics ^33^. Subpopulations of cancer cells with unique genomes exist in different times and places in one tumor ^34^. This heterogeneity is challenging for clinical diagnosis and causes the heterogeneous distribution of free drug or NDDS in tumor tissue ^35^. Thus, tumor heterogeneity should not be ignored when NDDSs are designed to increase the targeting ability and enhance the penetration. For instance, Kataoka *et al*. have discovered “dynamic vents” which undergo spontaneous and transient opening and closure within tumor microvessels ^36^. The eruption process can increase accumulation and retention of large NPs in sparsely vascularized tumors. By exploiting this natural phenomenon, investigators can develop new strategies to efficiently deliver and retain large NDDSs in deep tumor tissue. In addition, Chan *et al*. have reported that tumor pathophysiology and volume can significantly influence the targeting of NPs ^37^. As the Chinese proverb says, “Know yourself and know your enemy, you will win every war”. The more we know about the NDDS and the tumor characteristics, the better the therapeutic effect.

## Controllable release of the payload into the cytoplasm

Targeting and controllable release are the basic characteristics of NDDSs. Controllable release, which means that the loaded drugs are liberated in the right place and time, can significantly reduce administration times and avoid toxicity to other organs. In general, controllable release is facilitated mainly by external and internal stimulus-response strategies ^38, 39^. Internal stimulus-responsive NDDSs are sensitive to factors such as pH, redox status, and enzyme levels, which are often abnormal in tumor cells. Thus, the response efficiency is highly dependent on the specific biological conditions within the treated mouse model. Among the aforementioned strategies, enzyme-sensitive NDDSs have been enthusiastically explored because of their high selectivity and specificity ^40, 41^. However, the enzyme-triggered activation of NDDSs should be properly understood and the sensitivity of NDDSs *in vivo* should be measured because of the possibility of steric hindrance at the active site in the interior of NDDS. In contrast, external stimulus-responsive NDDSs are sensitive to external physical factors, including temperature, magnetic fields, ultrasound, light, and electrical fields. External stimulation offers more precise control profiles compared with internal stimulation, but the choice of “therapeutic window” is directly related to the outcome. It is still unclear whether these external physical factors can facilitate tumor metastasis and cause damage to the normal tissues. Meanwhile, the need for large external equipment to apply the stimulus also increases the difficulty of translation and application.

The therapeutic effect also has a close relationship with the controlled-release mechanism, which affects how the payload drug is transported from the interior of the NDDS to the cytoplasm. Thanks to mathematical models, some drug release mechanisms have been simulated. For example, the process of protein release from an NDDS has been accurately simulated with an established mathematical model by Shoichet *et al*. ^42^. Importantly, the simulated release process has also been confirmed by experiments with two proteins. The main controlled-release mechanisms that have been discovered so far include diffusion, osmosis, erosion, and dissolution ^43, 44^. Thus, computer simulation provides a powerful tool for us to better design NDDSs for controllable release and to understand the release process.

## Circumvention of tumor drug resistance

Drug resistance, including intrinsic resistance and acquired resistance, is the main reason for the failure of chemotherapy ^45^. The major mechanisms of multi-drug resistance (MDR) are decreased drug uptake, increased efflux of drugs, and changes in cell behavior ^46^. Among these, increased efflux of drugs mediated by ATP-binding cassette (ABC) transporters, such as P-glycoprotein (P-gp) or ABCB1, has a close relationship with MDR ^47^. New approaches have also been developed to tackle MDR in tumors. For instance, NPs mimicking *Salmonella* have been engineered by McCormick *et al*. ^48^. The *Salmonella* mimics were constructed from gold NPs coated with the *S. typhimurium type III* secreted effector protein SipA, which reduces the function of P-gp. The bacterial mimics efficiently reduced the P-gp level and increased the tumor sensitivity to doxorubicin. Meanwhile, Artzi *et al*. have developed an implantable hydrogel-embedded ON/OFF molecular nanoswitch probe to sense and overcome cancer MDR ^49^. Although NDDSs have successfully overcome MDR in some cases, the question remains whether NDDS-induced resistance will occur after administration of multiple doses. Also, as discussed above, tumor heterogeneity makes this problem more complex because it is unclear which subpopulations of cancer cells are actually responsible for the resistance. Nevertheless, NDDSs are a promising choice for reversing drug resistance.

## Nanoparticle-based drug delivery systems as a drug-like modulator for therapy

NDDSs have always been used as a vehicle to deliver therapeutic drugs or imaging agents. Their physicochemical characteristics are carefully optimized to meet the demand of delivery and targeting. Interestingly, the fact that their physicochemical properties can be modified means that NDDSs can also be applied as drug-like modulators, without any loaded cargo, to treat disease. For example, Overholtzer *et al*. have reported that ultrasmall poly(ethylene glycol)-coated silica NPs can inhibit tumor growth by inducing ferroptosis in starved cancer cells and in sensitive tumors ^50^. At the same time, a study by Daldrup-Link *et al*. reported that iron oxide NPs can induce pro-inflammatory macrophage polarization in tumor tissues to suppress tumor growth ^51^. The outcome was based only on the intrinsic therapeutic effect of pure NPs or material rather than loaded drug, indicating that the pure NPs also have therapeutic potential. The intrinsic therapeutic effects of other nanomaterials should be explored next. It will be worth investigating the modulatory effects of NDDSs on growth inhibition, metastasis, and recurrence through regulation of tumor metabolism, signal transduction, ion transport, and other biological processes that are common in tumors. However, it is important to note that the potential toxicity of drug-like modulators to healthy organs should not be ignored, even though they may have a therapeutic effect on abnormal tissues.

## What do we need to do in the future?

**Figure 1.**
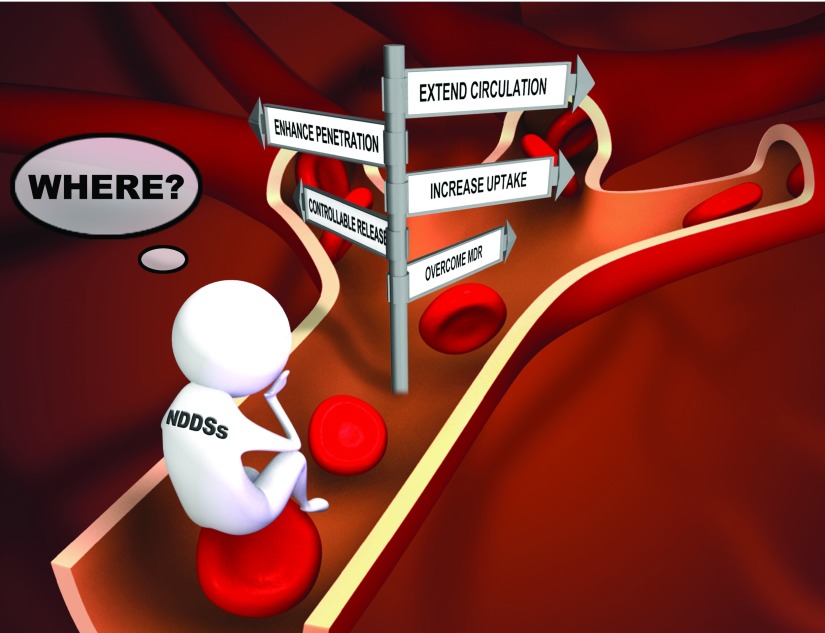
Nanoparticle-based drug delivery systems: What can they really do and what should they do *in vivo*?

NDDSs have been designed to kill cancer cells by using the strategies discussed above, and their *in vivo* behavior has been partly investigated in mouse models. In fact, an organism is a complex system and NDDSs encounter different biological milieus in different parts of the body. Therefore, the properties of NDDS will change dynamically during the delivery journey *in vivo*. Unfortunately, NDDSs may lose the ability to execute the tactics designed by the “coach” (scientist). Although scientists have developed many smart and complex NDDSs, these NDDSs may be too complicated for clinical trials or therapeutic use. Vigilance is necessary to ensure that a “multi-functional” NDDS is not a “non-functional” NDDS in which the overall effectiveness is severely compromised by the weakest function in the system. Thus, further work is still needed to answer the question “What can NDDSs really do *in vivo*?” ( Figure 1) and improve the translation of nanomedicines.

This problem may be solved in the following ways. Firstly, the fate of NDDSs, such as their integrity, surface characteristics, pharmacokinetics, biodistribution, and immunological effects, needs detailed tracing and analysis ^52– 54^. Advanced technologies and methods are essential for this challenging exploration. Secondly, there should be a normative evaluation framework to assess the efficiency of NDDSs, and rational animal models, such as organs-on-a-chip systems, should be established instead of just relying on the tumor size or survival curves in mouse models ^55^. The 5R framework (AstraZeneca’s 5R principle: right target/efficacy, right tissue/exposure, right safety, right patient, and right commercial potential) may be of beneficial guidance ^56^. It is equally important to combine the skills of chemists, mathematicians, biologists, and medical scientists to design clinically valuable NDDSs. Understanding the heterogeneity and biological nature of the tumor will really help us create NDDSs which may meet the expected treatment efficiency. In addition, we should pay more attention to structurally simple and reproducibly synthesized NDDSs because these have the greatest potential to reach the patient. Finally, it is important for us to keep in mind that we should constantly rethink what we are doing now and what we need to do in the future.

## Conclusions

NDDSs provide a flexible and versatile platform for tumor therapy. Given their longevity and targeting, NDDSs can efficiently penetrate tissue and controllably release their payload into the cytoplasm. Moreover, NDDSs can be used to overcome tumor drug resistance. In particular, the intrinsic therapeutic effects of pure NPs can be regarded as a new therapeutic strategy. However, our understanding of what NDDSs can really do is still limited *in vivo*, and translation of NDDSs is challenging. In summary, NDDSs are a promising choice for tumor therapy but many questions still need to be answered for their effective clinical translation.

## Abbreviations

ABC, ATP-binding cassette; EPR, enhanced permeability and retention; MDR, multi-drug resistance; MPS, mononuclear phagocyte system; NDDS, nanoparticle-based drug delivery system; NP, nanoparticle; P-gp, P-glycoprotein.
